# Relationships among Parvalbumin-Immunoreactive Neuron Density, Phase-Locked Gamma Oscillations, and Autistic/Schizophrenic Symptoms in PDGFR-β Knock-Out and Control Mice

**DOI:** 10.1371/journal.pone.0119258

**Published:** 2015-03-24

**Authors:** Tomoya Nakamura, Jumpei Matsumoto, Yusaku Takamura, Yoko Ishii, Masakiyo Sasahara, Taketoshi Ono, Hisao Nishijo

**Affiliations:** 1 System Emotional Science, Graduate School of Medicine and Pharmaceutical Sciences, University of Toyama, Sugitani 2630, Toyama, Japan; 2 Department of Pathology, Graduate School of Medicine and Pharmaceutical Sciences, University of Toyama, Sugitani 2630, Toyama, Japan; Tokyo Metropolitan Institute of Medical Science, JAPAN

## Abstract

Cognitive deficits and negative symptoms are important therapeutic targets for schizophrenia and autism disorders. Although reduction of phase-locked gamma oscillation has been suggested to be a result of reduced parvalbumin-immunoreactive (putatively, GABAergic) neurons, no direct correlations between these have been established in these disorders. In the present study, we investigated such relationships during pharmacological treatment with a newly synthesized drug, T-817MA, which displays neuroprotective and neurotrophic effects. In this study, we used platelet-derived growth factor receptor-β gene knockout (PDGFR-β KO) mice as an animal model of schizophrenia and autism. These mutant mice display a reduction in social behaviors; deficits in prepulse inhibition (PPI); reduced levels of parvalbumin-immunoreactive neurons in the medical prefrontal cortex, hippocampus, amygdala, and superior colliculus; and a deficit in of auditory phase-locked gamma oscillations. We found that oral administration of T-817MA ameliorated all these symptoms in the PDGFR-β KO mice. Furthermore, phase-locked gamma oscillations were significantly correlated with the density of parvalbumin-immunoreactive neurons, which was, in turn, correlated with PPI and behavioral parameters. These findings suggest that recovery of parvalbumin-immunoreactive neurons by pharmacological intervention relieved the reduction of phase-locked gamma oscillations and, consequently, ameliorated PPI and social behavioral deficits. Thus, our findings suggest that phase-locked gamma oscillations could be a useful physiological biomarker for abnormality of parvalbumin-immunoreactive neurons that may induce cognitive deficits and negative symptoms of schizophrenia and autism, as well as of effective pharmacological interventions in both humans and experimental animals.

## Introduction

Emerging evidence from EEG/MEG studies indicates that abnormal phase-locked gamma range (26–70 Hz) synchrony could be a biomarker reflecting core pathophysiological features of schizophrenia and autism with various cognitive and social abnormalities [1,2]. Gamma-band activity is generated through feedback inhibition on pyramidal neurons by synaptically and electrically connected (gap junction) networks of fast-spiking interneurons expressing the calcium-binding protein parvalbumin [3,4]. Consistent with deficits of gamma-band activity, parvalbumin-immunoreactive neuron density has been shown to be reduced in patients with schizophrenia and autism, as wells as in the brain of animal models of these diseases [5–7]. These findings suggest that deficits of parvalbumin-immunoreactive neurons and gamma-band activity might be neural biomarkers of the shared cognitive deficits and negative symptoms associated with schizophrenia and autism, common between both human and animals. However, no previous study has investigated the direct relationships among these important neurophysiological, immunohistological, and behavioral parameters *in vivo*.

Various genetic animal models of neuropsychiatric disorders have been used to develop pharmacological treatments. Schizophrenic patients display 3 major symptoms (positive symptoms, negative symptoms, and cognitive deficits), which were modeled as animal models of neuropsychiatric disorders [8–10]. Autistic patients also display similar deficits (social deficits similar to negative symptoms and cognitive deficits), which were also modeled in animal models of autism [11,12]. On the other hand, polymorphisms and haplotypes of platelet-derived growth factor (PDGF) receptor β (PDGFR-β) are associated with schizophrenia [13], and serum PDGF levels are altered in boys with autism [14]. Mice with a knockout of the PDGFR-β gene (PDGFR-β KO) display cognitive deficits (e.g., deficits in prepulse inhibition [PPI], fear conditioning, and spatial memory) and behavioral changes related to negative symptoms (impeded social interaction, depressive-like immobility) of schizophrenia and autism as well as reduction of phase-locked gamma-band activity and parvalbumin-immunoreactive neurons [15]. PDGFR-β KO mice also displayed locomotor hyperactivity [15], which is one of behavioral phenotypes of animal models of autism and schizophrenia [16–18]. These findings suggest that PDGFR-β KO mice fulfill face (symptom homology) and construct (similar neurobiological mechanisms) validity of an animal model of schizophrenia and autism.

T-817MA (1-{3-[2-(1-benzothiophen-5-yl) ethoxy] propyl}-3-azetidinol maleate) was developed as a therapeutic agent for neurodegenerative disorders. It exerts neuroprotective effects against H_2_O_2_-induced oxidative stress [19], promotes neurite outgrowth *in vitro* by increasing levels of neurotrophic factors [19], and ameliorates cognitive impairments in an animal model of Alzheimer’s disease [20,21]. In the present study, we investigated the effects of T-817MA administration on PDGFR-β KO induced-dysfunction of parvalbumin-immunoreactive neurons, reduction of phase-locked gamma oscillations, and deficits in social interaction and PPI. Since these immunohistological, neurophysiological, and behavioral parameters could be associated with one another [2,22,23], we also analyzed relations among these parameters in order to determine the usefulness of phase-locked gamma oscillations as a possible biomarker for pharmacological intervention in both human and animals.

## Materials and Methods

### Animals

All mice were housed in individual cages in a temperature-controlled environment with a 12/12-h light/dark cycle (lights were turned on at 08:00, and off at 20:00). Food and water were supplied ad libitum. Mice (8 weeks old at the start of the experiment) were handled for three consecutive days before the start of experiments. All experimental protocols were performed in accordance with the guidelines for care and use of laboratory animals approved by the University of Toyama and the National Institutes of Health’s Guide for the Care and Use of Laboratory Animals, and approved by the Ethics Committee for Animal Experiments at the University of Toyama (License number: S-2009MED-9). All surgery was performed under avertin anesthesia, and all efforts were made to minimize suffering.

### Generation of conditional PDGFR-β KO mice

The Cre/loxP system was used to develop conditional PDGFR-β KO mutants [24]. Briefly, we crossed mutant mice harboring the PDGFR-β floxed allele with those expressing Cre recombinase under the control of the nestin promoter and enhancer that express in neurons from embryonic day 10.5, as previously described [25]. Before this cross, both mutant mice harboring floxed PDGFR-β and nestin-Cre^+^ were outbred with mice of the C57BL/6J (B6/J) strain for 14 generations in order to replace the genetic background of our mutant mice with that of the B6/J strain. PCR of tail DNA using oligonucleotide primers pairs for floxed PDGFR-β and for the Cre transgene was performed to confirm genotypes (see Ishii et al. [25]). Genotyping was confirmed by western blot analysis of the total lysates obtained from adult mouse brains to show that PDGFR-β expression had decreased to undetectable levels in PDGFR-β KO mice at cerebral cortex, hippocampus, mid brain, rhombencephalon and cerebellum [25]. In the present study, the following two types of male mice were used: mice with the Cre transgene and floxed PDGFR-β (PDGFR-β KO mice) and mice without the Cre transgene but with floxed PDGFR-β (control mice).

### Surgery for neurophysiological recordings

Under avertin anesthesia (187.5 mg/kg, i.p.), the mice underwent surgical implantation of two screws (M1.4mm 3 mm) in the skull over the frontal cortex (AP: +1.7, L: −1.3 mm relative to bregma) and cerebellum (AP: −6.3, L: +1.5 mm) [15]. These screws later served as differential EEG electrodes (impedance, 0.4kΩ at 1kHz) when a connector for the wires was joined to the screws and attached to the skull using cranioplastic. Thus, we recorded auditory evoked potentials from the dorsal skull, in which multiple subcortical regions rather than the auditory cortex are suggested to be origins of those potentials [26, 27].

### Oral administration of T-817MA and experimental schedule

T-817MA was synthesized by Toyama Chemical Co., Ltd. (Toyama, Japan), and was dissolved in distilled water (DW) to the concentration of 1.0 mg/ml that was of reference to Kawasaki et al. [28]. After recovery from surgery (1 week), T-817MA (10 mg kg^−1^ day^−1^) or vehicle DW (10 ml kg^−1^ day^−1^) were administered orally for every day between 12:00–14:00 for four weeks. Thus, the mice were assigned to one of the following four groups: control mice administered with DW (Cont-DW, n = 20), control mice administered with T-817MA (Cont-T817, n = 19), PDGFR-β KO mice administered with DW (KO-DW, n = 19), or PDGFR-β KO mice administered with T-817MA (KO-T817, n = 21). T-817MA can penetrate the blood–brain barrier, and after oral administration in rodents, its concentration in the cerebellum is about 7.5 times higher than that in the plasma [29]. After administration of DW or T-817MA for four weeks, the 4 experiments were performed in the following order; neurophysiological recording of auditory evoked potentials, social interaction test, and PPI test. After the PPI test, the mice were perfused for immunohistological investigation (see below for details).

### Neurophysiological recordings of auditory phase-locked gamma oscillations

Sensory evoked gamma oscillations are recorded from multiple brain regions, and suggested to be involved in sensory perceptual and cognitive processes (see reviews by Başar et al. [30, 31]). Since deficits in early auditory phase-locked gamma oscillation have been reported in schizophrenia [32], we analyzed auditory phase-locked gamma oscillations in PDGFR-β KO mice. Animals were put into a plastic recording box (235 × 185 × 125 mm), and EEG signals collected from the electrodes were amplified and band-pass filtered at 1.5–1000 Hz (3 dB corner, 6 dB octave/slope) using an amplifier (MEG-5200G, Nihon Kohden, Tokyo, Japan). The amplified signals were digitized at 20 kHz and stored using Spike2 (ver. 6) (Cambridge Electronic Design, Cambridge, UK). Auditory stimuli (9 kHz pure tone, 500 ms duration, 100 times) were produced using a sound generator (DPS-725T, DIA Medical System, Tokyo, Japan), amplified to 85 dB at an interval of 3 s with a background noise of 50 dB, and delivered using a speaker. The speaker was located in front of the mice (30 cm away from the heads).

EEG data were downsampled to 1 kHz using Spike2 and analyzed by EEGLAB (Schwartz Center for Computational Neuroscience). One hundred single-trial epochs, ranging between −1.1 to 1.5 s from tone onset, were extracted from continuous EEGs. To indicate gamma oscillation, auditory evoked potentials in individual trials were digitally band-pass filtered between 26.4–67.1 Hz using butter worth filters (12dB/oct slope). Power spectrum density was computed using the periodogram method (500 frequencies between 1 and 100Hz). Total power was calculated for the time frequency window between 26.4–67.1 Hz. Then, the mean total power was compared among the four groups of mice.

Intertrial coherence (ITC) reflecting phase-locked synchronization (6–120 Hz) across individual trials, which is independent of the signal amplitude, was then computed between −100 to +100 ms from the tone onset at 1-kHz sampling [33]; ITC ranged from 0 (non–phase-locked, random activity) to 1 (fully phase-locked across individual trials). We then computed mean ITC in gamma bands (26.4–67.1 Hz) at 1-kHz sampling. Finally, a peak value of mean gamma ITC between 0 and 100 ms from the tone onset was measured as the “peak-ITC” for each animal. Furthermore, we analyzed peak-ITC in narrow gamma band (30.4–67.1 Hz) in the same way.

### Social interaction test

The testing cage consisted of a white plastic box (38.5 × 22.5 × 20 cm) where individual mice were allowed to acclimate for 20 min prior to testing onset. Pairs of mice from the same group were then placed in opposite corners of the box, and their activity was recorded using an overhead CCD camera for 30 min. Durations of social activities (e.g., proximity behavior, approaching and leaving, following, social sniffing, and active and passive contact) were automatically analyzed using the SocialScan program (CleverSys Inc., Reston, VA, USA). The plastic box was wiped with 70% ethanol and air dried between each trial [15].

The detailed definition of social behaviors has been described in the previous study [15]. Briefly, social contacts were defined as inter-body distances between two mice less than 20 mm, and were divided into active and passive social contacts. When one mouse approached and actively contacted another mouse with specific criteria of a ratio of movement speed of two mice, the mouse’s behavior was considered active. The remaining contacts were considered passive (e.g., sitting or lying). Approaching was defined as such when one mouse approached other mouse with the specific criteria of direction, speed, and distance covered. Social sniffing was defined as such when the distance between the noses of the sniffing mouse and the body of the other mouse being sniffed was less than 30 mm. Social leaving was defined as such when one mouse left from other mouse with the specific criteria of direction, speed, and distance covered. Social following was defined as such when one mouse followed other mouse with the specific criteria of movements of the two mice and distance between the two mice. Social mounting was defined as such when one mouse rode on other mouse, which was detected by the joint shape change of the two animals.

### Prepulse inhibition (PPI)

PPI can be easily measured in rodents in a manner almost identical to procedures used in humans [34,35]. PPI is an operational measure of sensorimotor gating, in which motor response to an abrupt, intense stimulus is inhibited by a weak lead stimulus [36], reflecting sensorimotor gating that is most widely used in animal models of schizophrenia and autism. The startle reflex measurement system (O’Hara & Co., Tokyo) was used to measure startle responses [15]. A test session began by placing a mouse in a plastic cylinder in a sound-attenuated chamber and leaving it undisturbed for 10 min. The background white noise level in the chamber was 65 dB, and the prepulse sound (0, 70, 72, 74, 78, and 82 dB) was presented for 120 ms before the startle stimulus (120 dB white noise [40 ms], main pulse) was provided. Each test session was composed of 36 trials with six blocks of the six prepulses/main pulse combination types presented in a pseudorandom order, such that each combination type was presented once within a block. The average intertrial interval was 15 s (range, 10–20 s). Startle responses were recorded for 140 ms (measuring the response every 1 ms), starting with the onset of the prepulse stimulus. These responses were corrected for the body weight of each mouse. The PPI percentage was then calculated using the following formula: ([startle amplitude in trials without prepulse] – [startle amplitude in trials with prepulse])/[startle amplitude in trials without prepulse] × 100.

### Immunohistochemistry

Under deep anesthesia with sodium pentobarbital (50 mg/kg body weight, i.p.), the mice were transcardially perfused with heparinized saline (0.9% w/v NaCl), followed by 4% paraformaldehyde dissolved in 0.1 M phosphate buffer (PB) (pH 7.4). After perfusion, the brains were removed from the skull, coronally cut into small blocks, and postfixed in 4% paraformaldehyde overnight. Fixed brain blocks were then cryoprotected in 30% sucrose dissolved in 0.1 M PB and frozen in dry ice. Next, 40-μm–thick sections were cut, placed in 0.01 M phosphate buffer saline (PBS), transferred into an antifreeze solution (25% ethylene glycol, 25% glycerin, and 50% 0.1 M PB), and then stored at −20°C until immunohistochemical staining was performed.

Five serial sections were collected for every 200 μm, with one used for parvalbumin immunocytochemistry, one for Nissl staining with cresyl violet, and three preserved for further use. Sections were stained with mouse monoclonal anti-parvalbumin antibodies (1:10,000 dilution in 1% horse serum PBS, Sigma, St. Louis, MO, USA) using the labeling protocol described in our previous study (see Nguyen et al. [15]). Negative control sections were produced by omitting the primary antibody, and no reaction product was observed in any of the control sections.

### Stereological analysis of parvalbumin-immunoreactive neurons

Images of the sections were obtained with an all-in-one fluorescence microscope system (BZ-9000, Keyence Corporation, Osaka, Japan). Using the brain atlas produced by Hof et al. [37] as reference, we counted immunoreactive cells in each of the three sections anatomically matched to the adjacent Nissl-stained sections [37] located at +1.1, +1.26, +1.42 mm to the anterior-to-posterior (AP) level from bregma in the medial prefrontal cortex (mPFC), including the medial orbital cortex, cingulate cortex area, prelimbic cortex, infralimbic cortex, and dorsal peduncular cortex. In the amygdala, we counted stained cells in the basolateral amygdala (BLA), including the lateral, basal, and accessory basal nuclei, in four sections at −1.44, −1.6, −1.76, and −1.92 mm AP. In the hippocampus and superior colliculus (SC), stained cells were counted in the five (−1.6, −1.76, −1.92, −2.08, −2.24 mm AP) and seven (−3.8, −3.96, −4.12, −4.28, −4.44, −4.6, −4.76 mm AP) sections, respectively.

Parvalbumin-immunoreactive neurons were counted using an optical dissector, an unbiased stereological technique [38]. Systematic sampling of the brain was performed by randomly translating a grid with 580.08 × 695.58-μm (mPFC), 251.71 × 337.00-μm (BLA), 767.50 × 408.59-μm (hippocampus), and 390.89 × 366.56-μm (SC) rectangles onto the section of interest using a stereology software (Stereo Investigator v.7.53.1, MicroBrightField, Williston, VT). Each intersection represented a sample site where 200 × 200-μm (mPFC, BLA, hippocampus) and 100 × 100-μm (SC) counting frames with exclusion lines [39, 40] were then applied. All randomly distributed, computer-generated sample sites were then examined using a 20× objective. Only parvalbumin-immunoreactive cell bodies falling within the counting frame without contact with the exclusion lines were enumerated. Objects seen in the counting frame were counted only if they came into focus within a predetermined 5-μm–thick optical dissector positioned 2 μm below the surface of the mounted section as indicated by the Z-axis microcator. Parvalbumin-immunoreactive cell density was estimated in each brain area of each animal using the stereology software.

### Statistical data analysis

Quantitative data were expressed as means ± SEM. Data were analyzed by two-way or three-way ANOVAs followed by the Bonferroni test. The statistical significance level was set at p < 0.05. Correlations among peak-ITC, parvalbumin-immunoreactive neuronal density, and behavioral parameters were analyzed by Pearson’s correlation coefficient. All statistical data processing was performed using the Statistical Package for the Social Sciences (SPSS) version 19.0 (SPSS Inc., Chicago, IL).

## Results

### PPI

**Fig 1:**
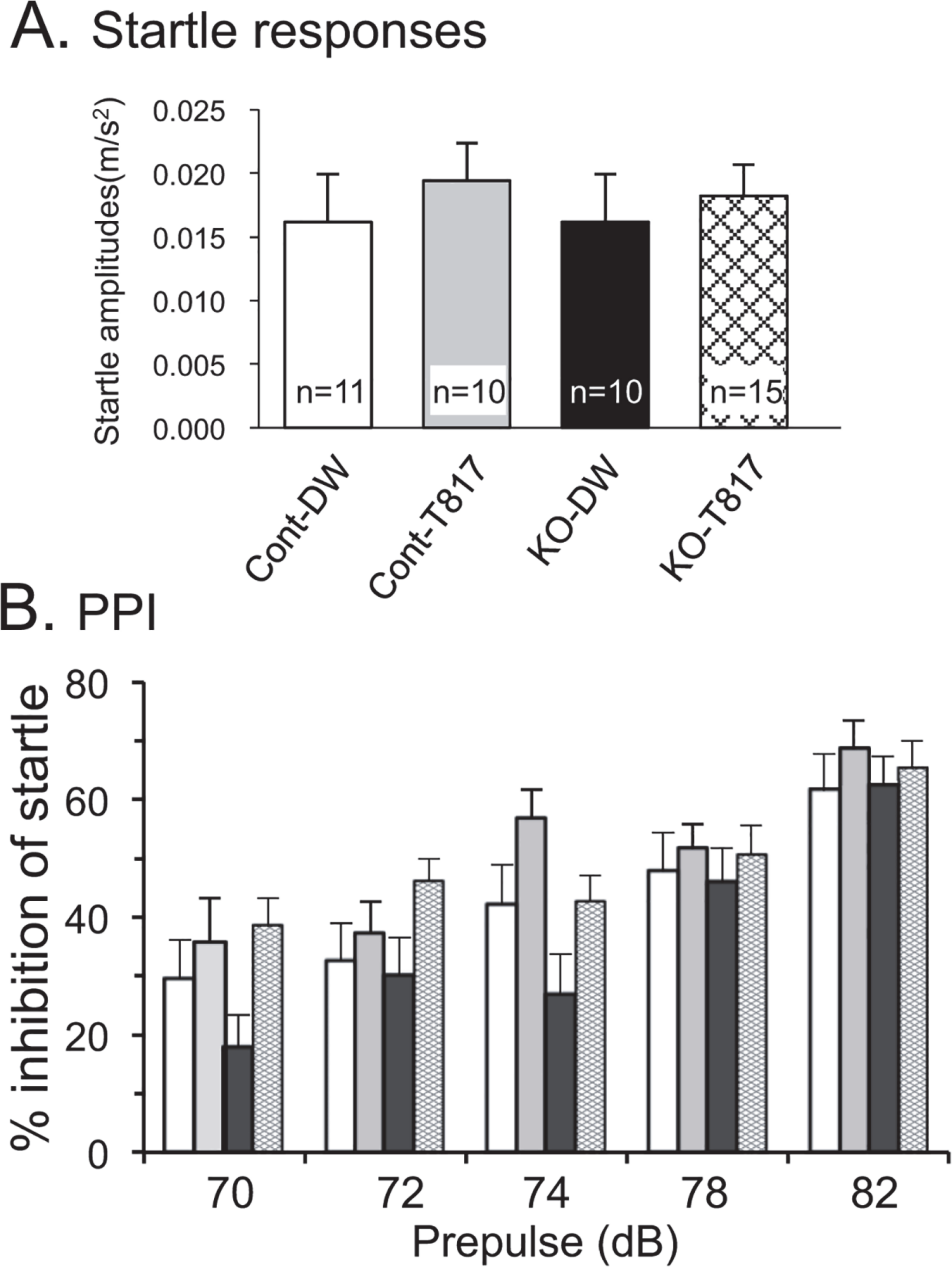
T-817MA ameliorated PPI deficits in PDGFR-β KO mice. (A) Acoustic startle amplitudes measured in trials without a prepulse. No significant differences were observed in the acoustic startle amplitudes among the four groups of mice. Values indicate the mean ± SE. (B) PPI (% inhibition) at five different prepulse intensities (70, 72, 74, 78, and 82 dB). Statistical results by three-way ANOVAs indicated significant main effects of genotype and treatment. Cont-DW, control mice with distilled water (DW); Cont-T817, control mice with T-817MA; KO-DW, PDGFR-β KO mice with DW; KO-T817, PDGFR-β KO mice with T-817MA.

Fig. 1A shows a comparison of startle responses to main pulses without a prepulse. Statistical results by two-way ANOVA indicated that there was no significant main effect or interaction (data not shown). Fig. 1B shows PPI results of the four mice groups. Statistical analysis by a three-way ANOVA (genotype × treatment × prepulse intensity) indicated that there were significant main effects of genotype [F(1, 210) = 8.886; p<0.005] and treatment [F(1, 210) = 19.860; p<0.005], without significant interaction among genotype, treatment, and prepulse intensity [F(4, 210) = 0.446; p = 0.775] and between genotype and treatment [F(1, 210) = 1.343; p = 0.248]. These findings indicated that KO mice displayed deficits in sensorimotor gating, which were ameliorated by T-817MA although the effects were not specific to the KO mice. Furthermore, when the analysis was confined to the data in the 74 dB prepulse condition, statistical analysis by one-way ANOVA indicated that there were significant group differences [F(3, 42) = 7.633; p<0.01]. Bonferroni post-hoc comparisons revealed that sensory motor gating was significantly impaired in the KO-DW; %inhibition was significantly smaller in the KO-DW than the Cont-DW mice (p<0.05), Cont-T817 (p<0.01), and KO-T817 mice (p<0.05). These findings indicated that the KO mice displayed deficits in sensorimotor gating, which were ameliorated by T-817MA.

### Social interaction

**Fig 2:**
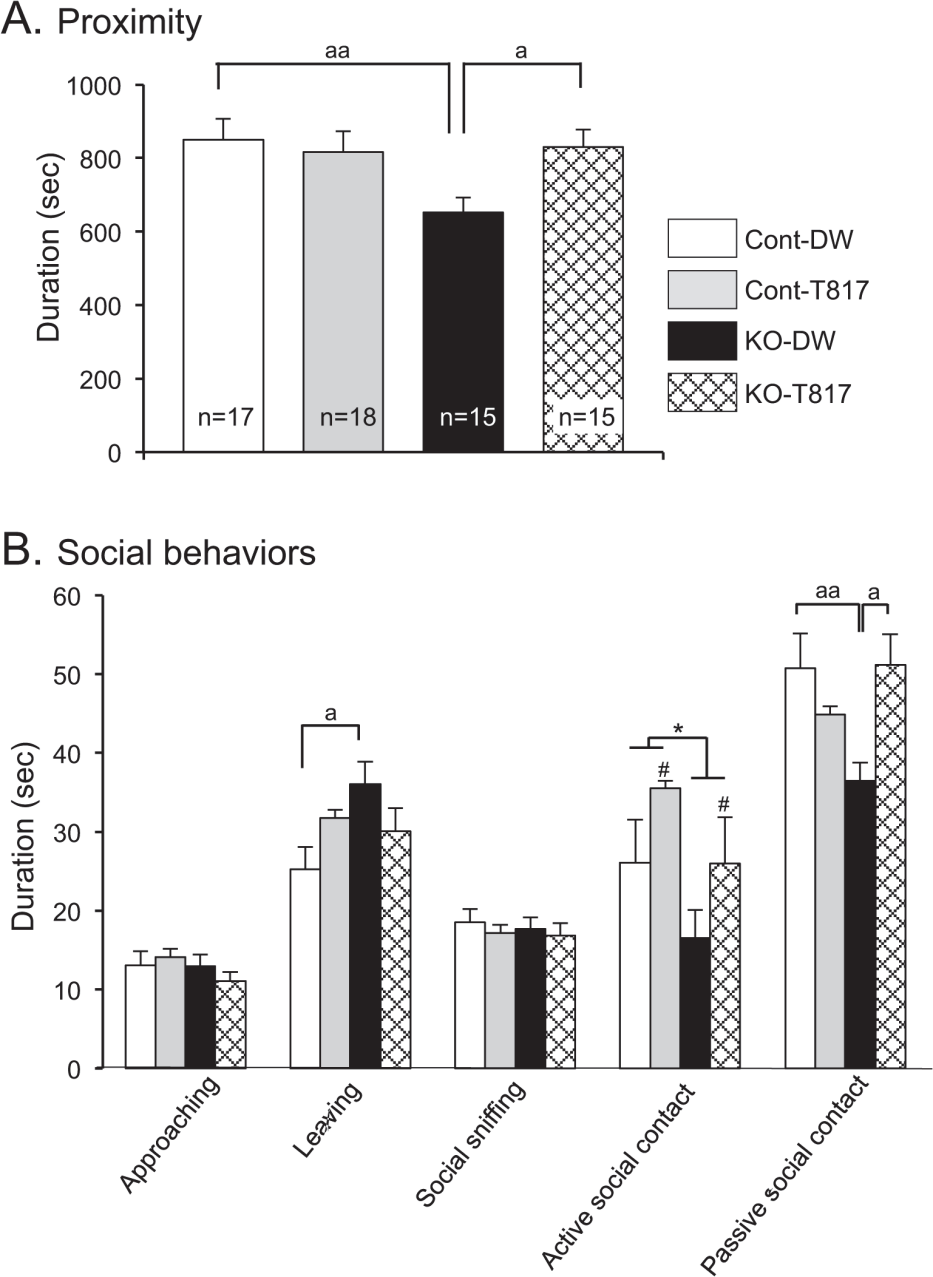
T-817MA ameliorated social interaction deficits in PDGFR-β KO mice. (A, B) Durations of each social behavior are indicated. a, p<0.05; aa, p<0.01 (Bonferroni test); *, p<0.05 (main effect of genotype); #, p<0.05 (main effect of treatment).

Fig. 2 shows duration of proximity (A) and other social behaviors (B). Statistical comparison of each behavior by two-way ANOVA (genotype × treatment) indicated significant main effects of genotype [active social contact (B); F(1, 61) = 4.470, p<0.05] and treatment [active social contact (B); F(1, 61) = 4.405, p<0.05], and significant interactions between these two [Proximity (A); F(1, 61) = 4.234, p<0.05; Leaving (B); F(1, 61) = 4.278, p<0.05; Passive social contact (B); F(1, 61) = 7.496, p<0.01]. Bonferroni post-hoc comparisons revealed that the duration of affiliate social behaviors was shorter in the KO-DW than in the Cont-DW mice [Proximity (A), p<0.01; Passive social contact (B), p<0.01], which was ameliorated by T-817MA [Proximity (A), p<0.05; Passive social contact (B), p<0.05]. On the other hand, compared to the Cont-DW mice, the KO-DW mice showed significantly higher durations of leaving (p<0.05) (B). These findings suggest that duration of affiliate social interaction was lower and non-affiliate behavior (Leaving) was higher in the KO-DW mice, which was ameliorated by T-817MA. Video recordings were also manually analyzed and revealed no aggressive behaviors in any of the groups. On the other hand, there were no significant differences in frequencies of social behaviors (data not shown).

### Neurophysiological recordings

**Fig 3:**
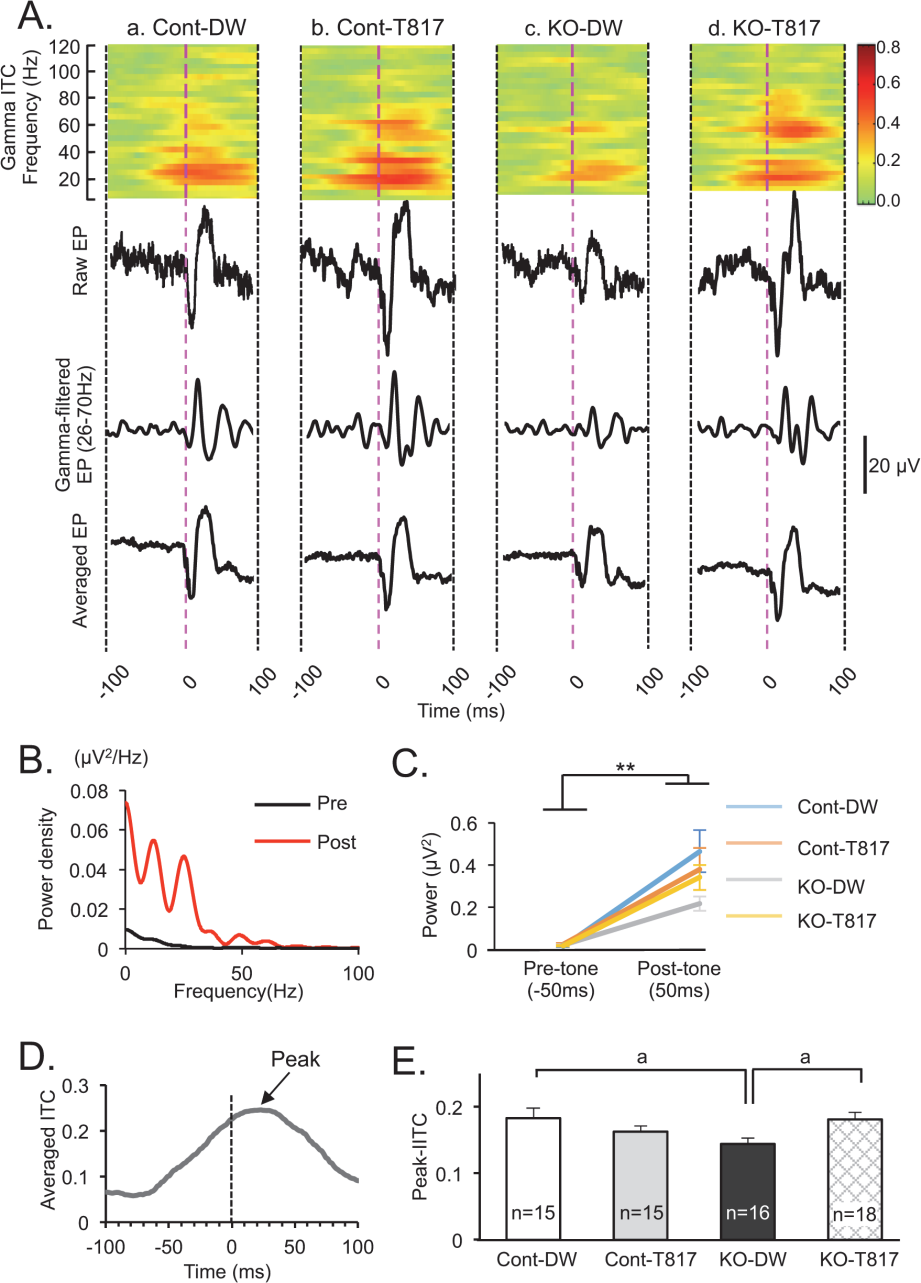
T-817MA ameliorated deficits of phase-locked gamma oscillations (peak-ITC, 26.4–67.1 Hz) in PDGFR-β KO mice. (A) Examples of ITCs (Gamma ITC), non-averaged evoked potentials in one trial (Raw EP), non-averaged gamma-filtered evoked potentials (Gamma-filtered EP) and averaged evoke potentials (Averaged EP) in the four groups of mice. (B) Power spectral density of the auditory evoked potentials during 50 ms before (Pre, black line) and after (Post, red line) the tone onset in the Cont-DW mouse shown in A. (C) Changes in mean total gamma power (26.4–67.1 Hz) of the evoked potentials during 50 ms before (Pre-tone) and after (Post-tone) the tone onset in Cont-Dw (blue line), Cont-T817 (orange line), KO-DW (grey line) and KO-T817 (yellow line). (D) Time course of mean ITC between 26.4–67.1 Hz for the Cont-DW mouse shown in Aa. The arrow indicates peak-ITC. (E) Comparison of peak-ITC among the four groups. Peak-ITC was significantly lower in PDGFR-β KO mice administered with distilled water (KO-DW) compared to control mice administered with distilled water (Cont-DW) and PDGFR-β KO mice administered with T-817MA (KO-T817). a, p<0.05 (Bonferroni test).

Fig. 3A illustrates examples of phase-locked gamma oscillations (Gamma-ITC), typical non-averaged auditory-evoked potentials in one trial (Raw EP), typical non-averaged gamma-filtered evoked potentials (Gamma-filtered EP) and averaged evoked potentials (Averaged EP) in the four groups. The data in the “Gamma-filtered EP” indicate that the auditory evoked potentials include gamma oscillation. ITC values at individual gamma frequencies ranged from 0.2 to 0.6, which are comparable to those in previous papers [41, 42, 18]. Among the four mice, the KO-DW mouse showed the lowest ITC in the gamma band (26.4–67.1 Hz) (Fig. 3Ac). Although the ITC was increased before the tone onset (Fig. 3A), the increases were not ascribed to accidental noisy EEG activity before the tone onset. First, the data in the “Averaged EP” indicate stable baselines before the tone onset. Second, we analyzed the power spectral density and total power before and after the tone onset; Fig. 3B shows auditory power spectral density of the evoked potentials during 50 ms before (black line, Pre) and after (red line, Post) the tone onset in the Cont-DW mouse. Statistical comparison of total power in the gamma band (26.4–67.1 Hz) by three-way repeated measure ANOVA (genotype × treatment × time) indicated a significant main effect of time [F(1, 60) = 85.802; p<0.01] (Fig. 3C). The data indicate that spectral power before the tone onset was much smaller, suggesting that the ITC increases before the tone onset (see below) were not ascribed to noisy EEG activities, consistent with the previous studies [43, 44, 45, 46].

Fig. 3D shows the time course of mean ITC between 26.4–67.1 Hz in the Cont-DW mouse shown in Fig. 3A, peaking at around +30 ms. Statistical comparison of peak-ITC in the gamma band by a two-way ANOVA indicated a significant interaction between genotype and treatment [F(1, 60) = 7.091; p<0.05] (Fig. 3E). Bonferroni post-hoc tests indicated that peak- ITC was significantly lower in KO-DW mice than in Cont-DW and KO-T817 mice (p<0.05).

**Fig 4:**
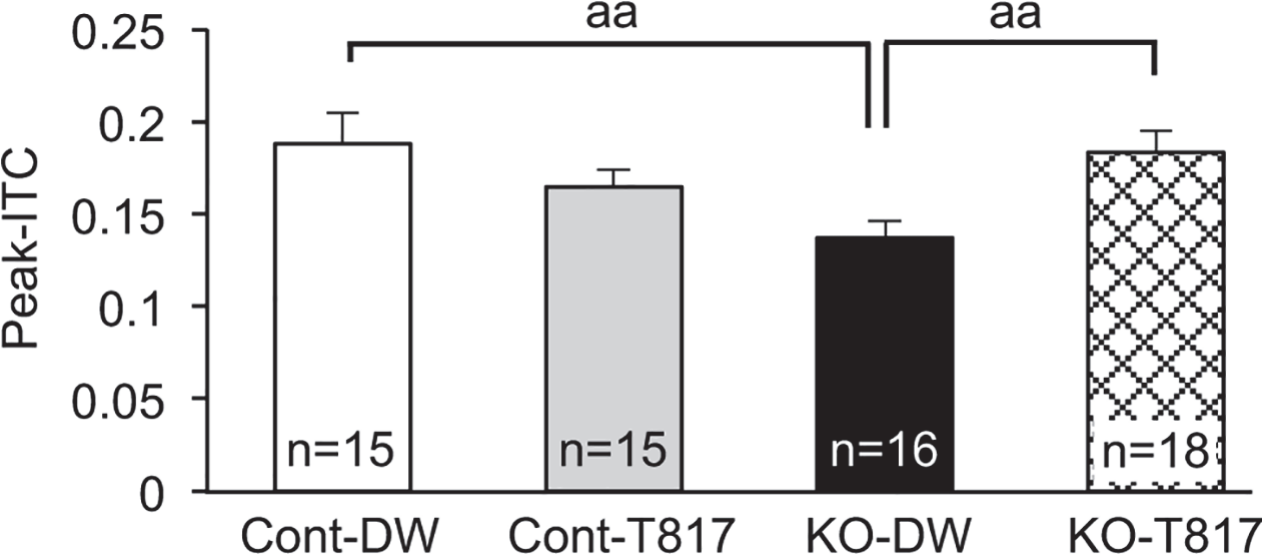
Comparison of peak-ITC (30.4–67.1 Hz) among the four groups. Peak-ITC was significantly lower in PDGFR-β KO mice administered with distilled water (KO-DW) compared to control mice administered with distilled water (Cont-DW) and PDGFR-β KO mice administered with T-817MA (KO-T817). aa, p<0.01 (Bonferroni test).

Furthermore, we analyzed peak-ITC in the theta (6.0 Hz), alpha (10.1 Hz) and beta (14.1–22.3 Hz) bands in the same way. Statistical comparison of peak-ITC in the theta and beta bands by a two-way ANOVA indicated that there was no significant main effect, nor significant interaction (data not shown). In the alpha band, there was only a significant main effect of genotype [F(1, 60) = 4.455; p<0.05], but no significant interaction [F(1. 60) = 2.665; p = 0.108]. Thus, effects of T-8717 MA were not observed in other frequency bands. In addition, we analyzed peak-ITC in the narrow gamma band (30.4–67.1 Hz) in the same way. Statistical comparison of peak-ITC by a two-way ANOVA indicated a significant interaction between genotype and treatment [F(1, 60) = 8.264; p<0.01] (Fig. 4). Bonferroni post-hoc tests indicated that peak-ITC was significantly lower in KO-DW mice than in Cont-DW and KO-T817 mice (p<0.01).

### Immunohistological analyses

**Fig 5:**
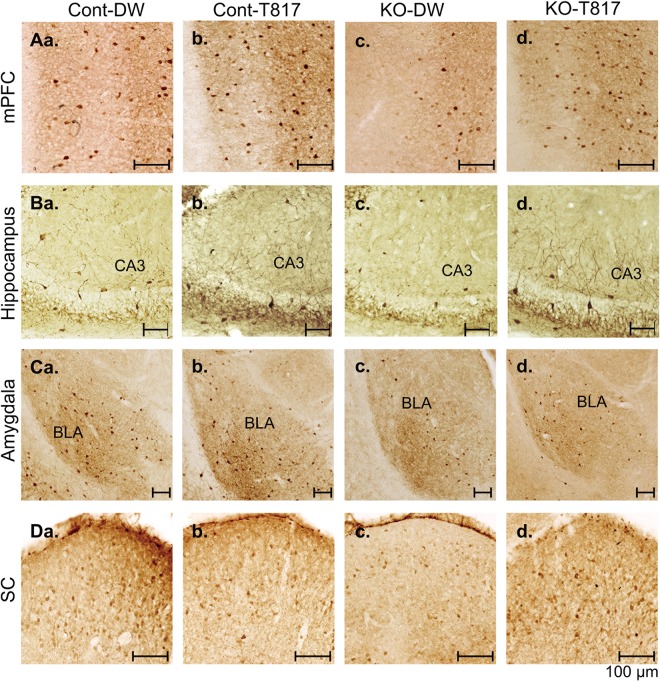
Photomicrographs of the medial prefrontal cortex (mPFC, A), CA3 subfield of the hippocampus (B), basolateral amygdala (BLA, C), and superior colliculus (SC, D) of the four groups of mice. Number of parvalbumin-immunoreactive neurons was decreased in PDGFR-**β** KO mice (KO-DW). Scale bar = 100 μm.

**Fig 6:**
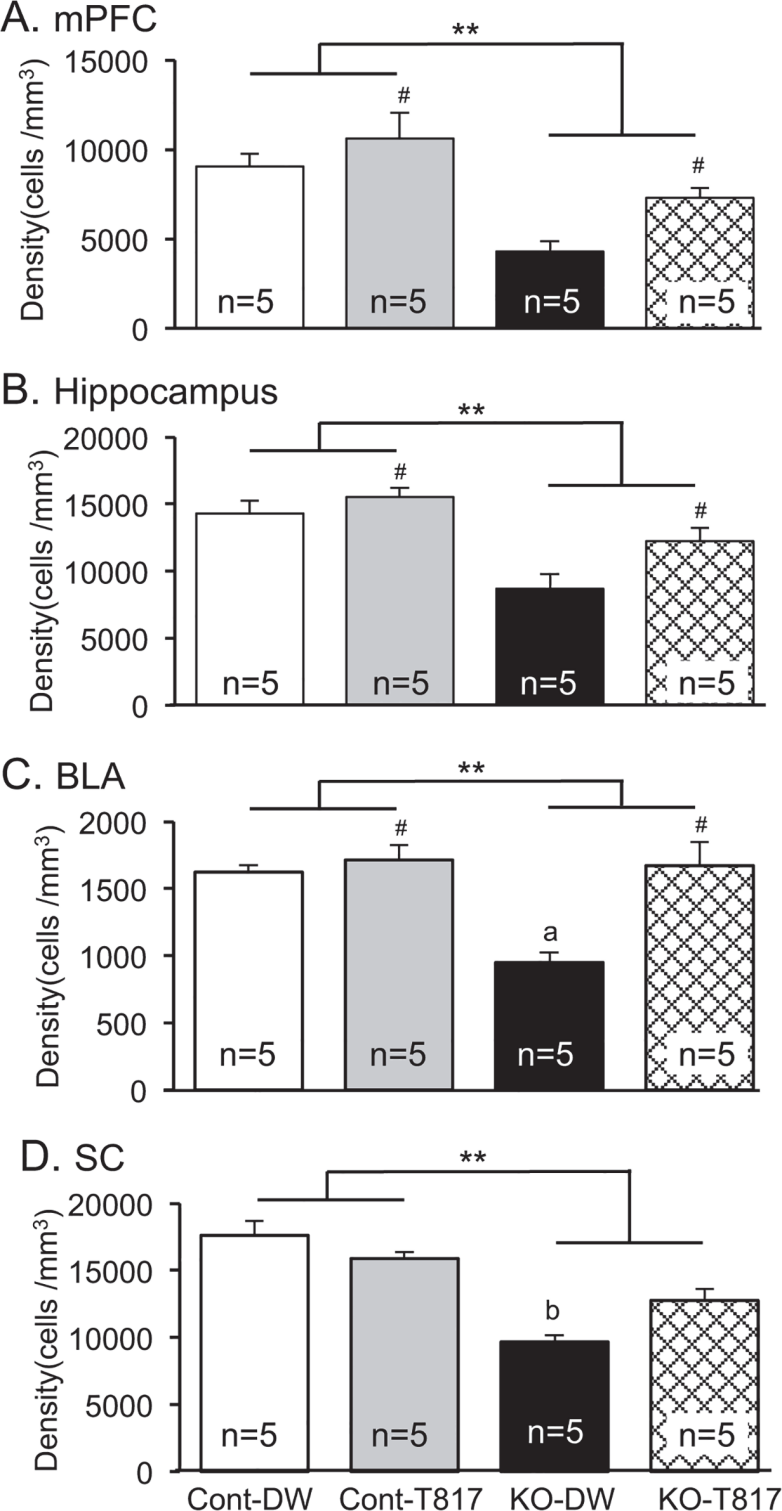
T-817MA ameliorated the decrease in density of parvalbumin-immunoreactive neurons in the mPFC (A), hippocampus (B), basolateral amygdala (BLA) (C), and superior colliculus (SC) (D) of PDGFR-β KO mice. **, p<0.01 (main effect of genotype); #, p<0.05 (main effect of treatment); a, significant difference between Cont-DW and KO-T817 in the BLA (Bonferroni test, p<0.01); b, significant difference between Cont-DW (Bonferroni test, p<0.01) and KO-T817 (Bonferroni test, p<0.05) in the SC.

Fig. 5 shows examples of parvalbumin-immunoreactive neurons in the mPFC (A), CA3 subfield of the hippocampus (B), basolateral amygdala (BLA) (C), and SC (D) for the four groups. A reduction in parvalbumin-immunoreactive neurons was observed in KO-DW mice (c) in comparison to other group mice (a, b, and d). Fig. 6 shows the mean density (cells/mm^3^) of parvalbumin-immunoreactive neurons in the mPFC (A), hippocampus (B), BLA (C), and SC (D). Statistical comparisons by two-way ANOVAs in each area indicated significant main effects of genotype in the mPFC [F(1, 16) = 20.102; p<0.01], hippocampus [F(1, 16) = 21.778; p < 0.01], BLA [F(1, 16) = 10.073; p<0.01], and SC [F(1, 16) = 51.466; p<0.01], and significant main effects of treatment in the mPFC [F(1, 16) = 6.446; p<0.05], hippocampus [F(1, 16) = 6.262; p<0.05], and BLA [F(1, 16) = 13.031; p<0.05]. Furthermore, there was a significant interaction in the BLA [F(1, 16) = 7.879; p<0.05] and SC [F(1, 16) = 7.879; p<0.05], and Bonferroni post-hoc tests revealed that cell density was significantly lower in the KO-DW than in the Cont-DW (BLA and SC, p<0.01) and KO-T817 mice (BLA, p<0.01; SC, p<0.05). These findings suggest that KO mice showed a decrease in parvalbumin-immunoreactive neurons, which was ameliorated by T-817MA.

### Relationships among the parameters

**Fig 7:**
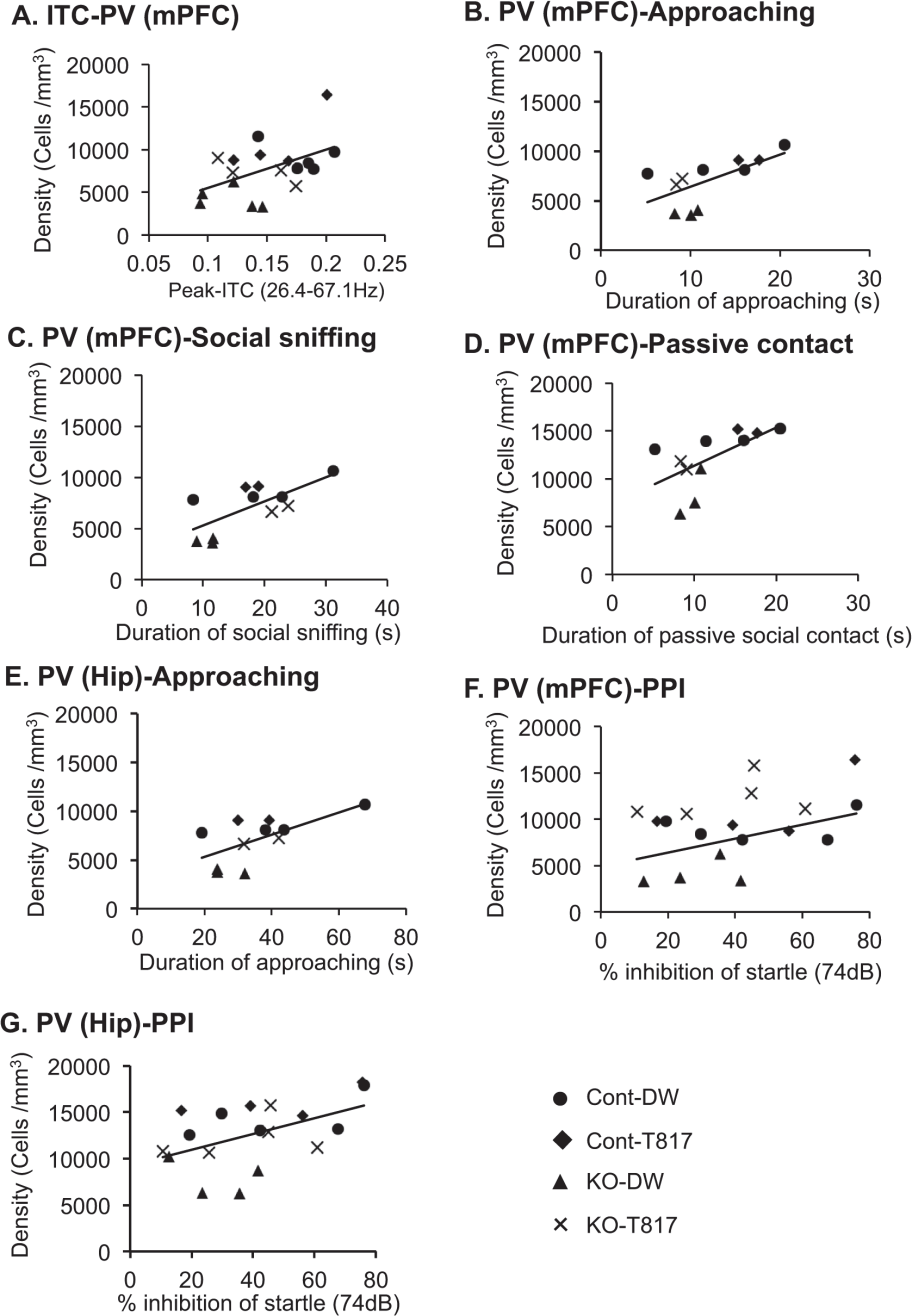
Significant correlations among neurophysiological, immunohistological, and behavioral parameters. (A) Significant correlation was observed between peak-ITC and parvalbumin-immunoreactive neuron density in the mPFC. (B-E) Significant relationships were observed between parvalbumin-immunoreactive neuron density in the mPFC and the duration of approaching (B), parvalbumin-immunoreactive neuron density in the mPFC and the duration of social sniffing (C), parvalbumin-immunoreactive neuron density in the mPFC and the duration of passive contact (D), parvalbumin-immunoreactive neuron density in the hippocampus (Hip) and the duration of approaching (E) (p<0.05). (F, G) Significant relationships were observed between parvalbumin-immunoreactive neuron density in the mPFC and % inhibition at 74 dB in the PPI test (F) and parvalbumin-immunoreactive neuron density in the hippocampus (Hip) and % inhibition at 74 dB in the PPI test (G) (p<0.05). Circles, Cont-DW; squares, Cont-T817; triangles, KO-DW; Crosses, KO-T817.

The relationship between the peak-ITC and the other parameters (cell density of parvalbumin-immunoreactive neurons and social behavior) were first analyzed. The results indicated that the peak-ITC was significantly correlated with immunoreactive cell density in the mPFC (Pearson’s correlation, r = 0.497, p<0.05; Cont-DW, n = 5; Cont-T817, n = 4; KO-DW, n = 5; KO-T817, n = 4) (Fig. 7A), and tended to be correlated in the hippocampus (r = 0.413; p = 0.089), BLA (r = 0.468; p = 0.050), and SC (r = 0.438; p = 0.069). It is noted that there was no significant correlation within each group, and the apparent correlation reflects the defects in KO-DW. On the other hand, there was a trend toward correlation between peak-ITC and the duration of proximity (Pearson’s correlation, r = 0.256; p = 0.072; Cont-DW, n = 8; Cont-T817, n = 11; KO-DW, n = 17; KO-T817, n = 14). Significant correlation was not observed between the peak-ITC and duration of any other social behavior (data not shown).

Further, immunoreactive cell density in the mPFC was significantly correlated with the duration of social behaviors (Pearson’s correlation; approaching, r = 0.640, p<0.05; social sniffing, r = 0.700, p<0.05; passive social contact, r = 0.636, p<0.05; Cont-DW, n = 4; Cont-T817, n = 2; KO-DW, n = 3; KO-T817, n = 2) (Fig. 7B, C, D). Cell density of parvalbumin-immunoreactive neurons in the hippocampus was correlated with the duration of approaching behavior (r = 0.619, p<0.05) (Fig. 7E) and tended to be correlated with the duration of sniffing behavior (r = 0.576, p = 0.064). Cell density in the BLA also tended to be correlated with the duration of sniffing behavior (r = 0.566, p = 0.069). Significant correlation was not observed between the cell density and duration of any other social behavior (data not shown).

In addition, immunoreactive cell density in the mPFC was significantly correlated with PPI parameters (% inhibition of startle using Pearson’s correlation; at 74 dB of prepulse r = 0.499, p<0.05 and at 78 dB of prepulse, r = 0.500, p<0.05; Cont-DW, n = 5; Cont-T817, n = 4; KO-DW, n = 4; KO-T817, n = 5 [see Fig. 7F for 74dB]), and there was a trend toward correlation at 82dB of prepulse, r = 0.464, p = 0.052. Furthermore, immunoreactive cell density in the hippocampus and PPI parameters were correlated at 74 dB of prepulse, r = 0.505, p<0.05 (Fig. 7G), and tended to be correlated at 78 dB of prepulse, r = 0.428, p = 0.076. Significant correlations were not observed between the cell density in any other region and PPI parameters (data not shown). Taken together, these findings suggest that density of parvalbumin-immunoreactive neurons were correlated with peak-ITC, PPI, and social behaviors.

## Discussion

### Effects on parvalbumin-immunoreactive neurons

In this study, density of parvalbumin-immunoreactive neurons was decreased in the mPFC, hippocampus, BLA, and SC of PDGFR-β KO mice. Since PDGF-B/PDGFR-β signal exerts neurotrophic effects on cultured GABAergic interneurons by increasing the expression of glutamic acid decarboxylase and the survival of these cells [47], a decrease in the density of parvalbumin-immunoreactive neurons might be attributed to the lack of the neurotrophic effects of PDGFR-β in PDGFR-β KO mice [15]. Furthermore, PDGFR-β exerts protective effects on neurons against various brain injuries and conditions, including oxidative stress [25, 48]. Oxidative stress has been proposed to induce loss and/or impairment of maturation in parvalbumin-immunoreactive neurons (see review by Behrens and Sejnowski. [49]). Thus, PDGFR-β deletion might increase superoxide levels or decrease neurotrophic effects, consequently resulting in decreased number of parvalbumin-immunoreactive neurons in PDGFR-β KO mice.

Using three-dimensional (stereological) cell counting, we observed that T-817MA administration relieved the PDGFR-β KO-induced reduction of parvalbumin-immunoreactive neuron density in the mPFC, hippocampus, BLA, and SC. This is consistent with the findings of a previous study that used a two-dimensional cell counting method and reported that T-817MA ameliorates the decrease in parvalbumin-immunoreactive neurons in the mPFC and hippocampus of rats neonatally administered with MK-801, an *N*-methyl-d-aspartate (NMDA) receptor antagonist [50]. Since T-817MA has been show to exert neurotrophic effects *in vitro*, have neuroprotective effects on Aβ- and oxidative stress-induced neurotoxicity, and suppress the decrease of antioxidant enzyme (glutathione) levels [19, 21], it is plausible that T-817MA may have neuroprotective effects against oxidative stress and/or neurotrophic effects for parvalbumin-immunoreactive neurons in PDGFR-β KO mice.

### Gamma oscillation abnormalities

In this study, T-817MA ameliorated reductions of phase-locked gamma oscillations in PDGFR-β KO mice. Gamma oscillations are bands of synchronous cortical population activity that can be seen in the electroencephalograms of humans and other mammals [51] during a variety of cognitive processes [52–54]. This synchronous activity is thought to consist of subsets of temporally coactive pyramidal cells [55] and diverse GABAergic interneurons [56]. In particular, parvalbumin-immunoreactive basket cells that connect pyramidal cell somata and proximal dendrites have been shown to synchronize the firing of postsynaptic pyramidal cells [57]. Because each parvalbumin-immunoreactive basket cell connects hundreds of pyramidal cells [58] via gap junctions and synapses [4] and also connects to other parvalbumin-immunoreactive basket cells via gap junctions [59] and synapses [60], they can fire synchronously at high frequencies, exerting powerful effects on pyramidal cells [56]. Furthermore, genetic disruption of excitatory synapses in parvalbumin-immunoreactive interneurons suppressed gamma oscillations in vitro [61], and stimulation and inhibition of parvalbumin-immunoreactive interneurons by optogenetics enhanced and suppressed gamma oscillations, respectively [3]. These findings suggest that the decreased gamma-band activity in PDGFR-β KO mice might be ascribed to the reduced number of parvalbumin-immunoreactive neurons, and that T-817MA ameliorated gamma oscillation reduction by restoring this neuronal population (see above). Consistent with this idea, the present study provides the first *in vivo* evidence of the correlation between phase-locked gamma oscillations in the mPFC and parvalbumin-immunoreactive neuron density.

### Social interaction abnormalities

We found that the duration of affiliate behaviors was decreased in PDGFR-β KO mice. Deficits in social interaction are core symptoms of schizophrenia as negative symptoms (i.e., little interest in social behavior or increased social isolation) [62] and the core symptoms of autism [63]. Additionally, impairments in GABAergic neurotransmission have been reported to be associated with social disorders [64]. Interestingly, in this study, the density of parvalbumin-immunoreactive neurons in the mPFC and hippocampus and peak gamma-ITC were correlated with various social behaviors. Gamma oscillations have also been reported to be critical for development of social communication and learning from others since gamma oscillations are elicited in the PFC by infant eye contact [22]. Previous studies reported that stimulation of parvalbumin-immunoreactive interneurons in the medial PFC by optogenetics reversed deficits in social behaviors induced by stimulation of pyramidal neurons [65], and inhibition of parvalbumin-immunoreactive interneurons in the medial PFC decreased gamma oscillation [3]. The data in these two studies suggest that gamma oscillation induced parvalbumin-immunoreactive interneurons in the medial PFC is important for social behaviors. Taken together, these data suggest that the behavioral abnormalities observed in PDGFR-β KO mice might be ascribed to the deficits in GABAergic neurotransmission and gamma oscillations due to reduced density of parvalbumin-immunoreactive neurons. In addition, we showed that T-817MA ameliorated the social interaction deficits in PDGFR-β KO mice. It is noted that main effect of treatment was significant in active social contact (Fig. 2B), suggesting that T-817MA similarly increased active social contact regardless of genotype. T-817MA similarly increased the number of parvalbumin-immunoreactive neurons regardless of genotype (Fig. 6) (i.e., significant main effect of treatment). The changes in active social contacts by T-817MA might reflect these similar changes in the number of parvalbumin-immunoreactive neurons. Thus, our findings strongly suggest that the ameliorating effects of T-817MA on social interaction deficits might be attributed to its effects on parvalbumin-immunoreactive neurons.

### Sensorimotor gating abnormalities

We found that sensorimotor gating was disturbed in PDGFR-β KO mice, which was improved by T-817MA. However, the differences in PPI between the Cont-DW and KO-DW mice were less evident in the present study compared with the previous study [15]. In the present study, to analyze the possible relationships among the results across the different experiments, the mice received electrode and connector implantation before the all experiments so that the experimental conditions except the treatment were the same across the experiments. This point is different from the previous study [15], which used intact animals. Startle responses were measured using the small cylinder, in which the animals were placed. Electrode and connector on the heads might affect startle responses in the small cylinder in the present study.

PPI is mediated through a neural system that includes the pontine brainstem and SC, which receive modulatory input from the nucleus accumbens innervated by the PFC, hippocampus, and amygdala [66, 67], and is mediated by various neurotransmitters including GABA [68–70]. Deficits in PPI have been suggested as a useful endophenotype for the diagnosis of schizophrenia [71] and its animal models [72]. Additionally, patients with autism and animal models of the disease also display disruptions in PPI [63, 73]. Both of these neuropsychiatric diseases are associated with abnormalities in the fronto-limbic system [74], where the density of putative inhibitory parvalbumin-immunoreactive neurons was found to be reduced in PDGFR-β KO mice in the present study. Furthermore, the density of parvalbumin-immunoreactive neurons in the mPFC and hippocampus was correlated with PPI. These findings suggest that PPI deficits in PDGFR-β KO mice were ascribed to decreases in parvalbumin-immunoreactive neuron densities in the fronto-limbic regions and ameliorated by T-817MA via an increase in the density of parvalbumin-immunoreactive neurons in these areas. On the other hand, there were no significant relations between gamma-ITC and PPI. However, previous studies reported that gamma oscillations induced by pre-pulse and main-pulse tones were correlated with PPI [75, 76]. Absence of significant correlation in the present study might be ascribed to our methods in which we analyzed gamma-ITC in response to auditory tones not used in the PPI task.

### Sites of action of T-817MA

It has been proposed that schizophrenia, autisms, and Alzheimer’s disease share common cellular substrates of pathogenesis; oxidative stress and synaptic dysfunction [77–80]. PDGFR-β deletion might increase vulnerability to oxidative stress in parvalbumin-positive (putatively GABAergic) neurons in the PDGFR-β KO mice [15], and T-817MA with anti-oxidant effects may reverse the changes due to PDGFR-β reduction (see above sections). T-817MA also attenuates amyloid-beta-induced neurotoxicity through its anti-oxidant effects in vitro [19]. Furthermore, PDGFR-β KO mice displayed synaptic dysfunction; deficits in learning and long-term potentiation (LTP), and a decrease in synaptic density [81]. Amyloid-β42 (Aβ42) also induced deficits in LTP, and T-817MA reversed Aβ42-induced deficits in LTP induction in vitro [82]. In addition, LTP requires axonal transport [83] and T-817MA prevented Aβ42- or tau-induced impairment of axonal transport [84, 85]. These findings suggest that cognitive and behavioral deficits in PDGFR-β KO mice might be ascribed to pathogeneses (oxidative stress and synaptic dysfunction) similar to schizophrenia and autism as well as Alzheimer’s disease, which T-817MA might attenuate. Further studies are required to investigate ameliorative mechanisms of T-817MA.

### Conclusions

Clinical studies suggest that abnormalities in phase-locked gamma oscillations could serve as biomarkers reflecting core pathophysiological features of schizophrenia and autism with various cognitive and social abnormalities. Additionally, animal studies suggest that parvalbumin-immunoreactive neurons might play a critical role in the generation of gamma oscillations (see Introduction). The present study indicated that oral administration of T-817MA to PDGFR-β KO mice ameliorated the reduced density of parvalbumin-immunoreactive neurons, restored regular phase-locked gamma oscillations, and improved social interactions and PPI deficits. Furthermore, this study provides the first evidence that the density of parvalbumin-immunoreactive neurons is significantly related to phase-locked gamma oscillations (gamma-ITC), the duration of social interaction, and PPI. These results suggest that ITC could be used as a common biomarker of these psychiatric diseases in both humans and animals as well as for measuring the effectiveness of pharmacological intervention. However, the numbers of data are not enough in some analyses (e.g., Fig. 7A, D, and E), and further studies are required to confirm the results. Lastly, we suggest that T-817MA could be a new candidate for the treatment of negative symptoms and cognitive deficits associated with schizophrenia and autism.

## Supporting Information

S1 Dataset The numerical data of the individual mice used in the figures. (XLSX)
